# NMDA Receptor Stimulation Induces Reversible Fission of the Neuronal Endoplasmic Reticulum

**DOI:** 10.1371/journal.pone.0005250

**Published:** 2009-04-21

**Authors:** Krzysztof Kucharz, Morten Krogh, Ai Na Ng, Håkan Toresson

**Affiliations:** 1 Laboratory for Experimental Brain Research, Wallenberg Neuroscience Centre, Department of Clinical Sciences Lund, Lund University, Lund, Sweden; 2 Computational Biology and Biological Physics, Department of Theoretical Physics, Lund University, Lund, Sweden; The Research Center of Neurobiology - Neurophysiology of Marseille, France

## Abstract

With few exceptions the endoplasmic reticulum (ER) is considered a continuous system of endomembranes within which proteins and ions can move. We have studied dynamic structural changes of the ER in hippocampal neurons in primary culture and organotypic slices. Fluorescence recovery after photobleaching (FRAP) was used to quantify and model ER structural dynamics. Ultrastructure was assessed by electron microscopy. In live cell imaging experiments we found that, under basal conditions, the ER of neuronal soma and dendrites was continuous. The smooth and uninterrupted appearance of the ER changed dramatically after glutamate stimulation. The ER fragmented into isolated vesicles in a rapid fission reaction that occurred prior to overt signs of neuronal damage. ER fission was found to be independent of ER calcium levels. Apart from glutamate, the calcium ionophore ionomycin was able to induce ER fission. The N-methyl, D-aspartate (NMDA) receptor antagonist MK-801 inhibited ER fission induced by glutamate as well as by ionomycin. Fission was not blocked by either ifenprodil or kinase inhibitors. Interestingly, sub-lethal NMDA receptor stimulation caused rapid ER fission followed by fusion. Hence, ER fission is not strictly associated with cellular damage or death. Our results thus demonstrate that neuronal ER structure is dynamically regulated with important consequences for protein mobility and ER luminal calcium tunneling.

## Introduction

Activation of glutamate receptors triggers a multitude of intracellular signaling pathways important for many aspects of CNS physiology and disease. Some of these signaling events terminate on the endoplasmic reticulum (ER) and are important for many aspects of brain function [1]–[8]. The ER is generally considered a continuous organelle and neuronal ER is no exception; even the ER found within dendritic spines is connected to the bulk of the ER in the dendrites and soma [9]–[12]. The continuity of the ER is important for its normal function as a calcium store as well as for its role in the secretory pathway. The continuous ER lumen permits calcium tunneling so the ER calcium channels can gate significant amounts of calcium in local domains upon stimulation and may also permit the propagation of signals over long distances [13], [14]. In the secretory pathway, ER continuity allows mature proteins to move to the specialized ER exit sites from which they are trafficked to the Golgi [15]–[18].

In spite of the vital importance of ER continuity it is known that under certain conditions the ER in some non-neuronal cell types can undergo dramatic changes in structure leading to loss of continuity. Such dramatically altered ER structure has been reported in living cells such as sea urchin [19] and starfish [20] eggs at fertilization, different non-neuronal cell lines [21]–[23] and lacrimal cells [24]. Fragmentation (hereafter also called fission) of the ER will most likely have significant effects on most, if not all, aspects of ER function. Importantly, it can be predicted that long-term fragmentation of the ER is likely to be incompatible with cellular survival. Hence, for the fragmentation of the ER described above to qualify as a physiologically relevant phenomenon, it should be balanced by a mechanism mediating fusion of ER vesicles. Such events where ER fission is followed by fusion has been convincingly shown in starfish eggs following fertilization-induced ER fission [20]. The fact that the ER in several cell types undergo fission after physiological stimuli prompted us to explore this question in neurons in primary culture as well as in cultured organotypic slices. We describe that, indeed, the neuronal ER is a dynamic organelle and that the fission machinery is controlled specifically by NMDA receptor activity.

## Materials and Methods

### Primary hippocampal neuronal cultures

Animals were handled in accordance with Swedish law under permits to HT (M197-07, M223-06). Dissected uteri from embryonic day 17 pregnant NMRI mice were transferred to ice cold PBS (Gibco). Hippocampal subdissection was done in ice cold HBSS (Gibco) with 4.17 mM NaHCO_3_ (Gibco). Tissue was cut with a fine spring scissors, gently disaggregated by triturating, washed twice with HBSS/NaHCO_3_ (pH 7.4) and transferred to Neurobasal medium supplemented with 2% B-27, 0.5 mM L-glutamine, 1× pen/strep (all from Gibco) and 25 µM glutamate (Fluka). Cells were plated at 4×10^5^ cells/ml in imaging 4 well chamber slides (Nunc) coated with 10 µg/ml Poly-D-lysine (Sigma) and 5 µg/ml laminin (Sigma). At day in vitro (DIV) 4, cells were transfected to express either EGFP and DsRed2-ER (RedER) (Clontech; 0.4 µg/ml and 0.6 µg/ml respectively) or DsRed2 and EGFP-ER (gift from Thomas Oertner, FMI, Basel) using Lipofectamine 2000 (Invitrogen) according to the manufacturer's instruction. Liposome-containing medium was replaced after 3–5 h by glutamate-free Neurobasal medium supplemented as above.

### Generation of Thy1-RedER transgenic mice

The transgene construct was built to express RedER and EGFP from the same transcript by internal ribosome entry site (IRES) mediated EGFP translation. Translation from the IRES was very weak, however, and the EGFP signal too low for imaging. The RedER gene derived from pDsRed2-ER was cloned into pIRES2-EGFP vector linearized with *Nhe*I – *Bgl*II. Both vectors were commercially available from Clontech. The RedER-IRES2-EGFP fragment derived using *Nhe*I/*Not*I sites was cloned into the pThy-1 vector linearized with *Xho*I (gift from Joshua R. Sanes; Washington University) [25].

### Slice cultures

Organotypic hippocampal slice cultures were prepared from 7-days old Thy-1 RedER transgenic mice as described [26]. Mice were decapitated and brains were transferred to ice-cold HBSS (Gibco) with 20 mM HEPES (Sigma), 6 mg/ml D-glucose (Sigma) and pen/strep (Gibco). Hippocampi were dissected and cut into 250 µm thick slices using a McIlwan Tissue Chopper. Slices were transferred to Millicell culture inserts (Millipore) in 24-well culture plates. Cultures were maintained at 35°C in 50% MEM (Gibco) with 25% heat inactivated horse serum (Gibco), 18% HBSS/NaHCO_3_ (pH 7.4) supplemented with 6 mg/ml D-glucose (Sigma), 2% B-27 (Gibco), 4 mM L-glutamine and pen/strep. After 7 days in vitro slices were grown in maintaining medium (as above) without B-27 supplement.

### Live cell imaging and FRAP analysis

Live cell imaging was performed with a Zeiss LSM 510 inverted confocal microscope system equipped with heating insert P and incubator S perfused with humidified air with CO_2_ (5%). Multi track mode was used and EGFP was excited with the 488 Argon laser line and the emitted light detected after a 505–530 band pass filter. DsRed2 was excited with the HeNe 543 laser and emission detected after a 560 long pass filter. Temperature was maintained at 37°C by heating the air and with a lens heater. Primary cultures were imaged in their growth medium by using the 63× N.A. 1.4 objective. Slices used for collecting images were cut out from their insert and placed in an imaging chamber, perfused with carbogen-bubbled artificial cerebrospinal fluid (NaCl: 119, KCl: 2.5, MgSO_4_: 1.3, NaH_2_PO_4_: 1, NaHCO_3_: 26, CaCl_2_: 2.5, glucose: 11 (numbers are concentration in mM), pH 7.4) and imaged with the 63× lens at 37°C. Images were exported from the Zeiss LSM software as projected images in TIFF or uncompressed JPEG format. Adjustment of images (in LSM or Adobe Photoshop) was limited to changing the contrast and brightness of the whole image. For FRAP recordings the 10× objective was used to analyze slices in their inserts placed on a glass-bottom petri dish with slice culture medium. For FRAP experiments fluorescence intensity was measured from a selected region of interest (ROI) on a dendrite for 30 scanning cycles (∼40 sec), bleached with 100% He/Ne laser intensity for 80 cycles (∼10 sec) and FRAP curve was recorded for up to 170 cycles (∼250 sec). The same ROI was used at every FRAP recording in the respective experiment. In addition to recording the RedER FRAP signal, for primary cultures, the EGFP signal was recorded in another channel in the multi track mode to serve as a control for fluctuations in the FRAP signal due to e.g. focal drift. Furthermore, a non-bleached ROI was always defined outside the dendrite, but adjacent to the dendrite ROI, to collect background signal (Fig. S2). The FRAP signal used for the analysis was defined as the ratio between the red and the green channel after subtracting the background in the two channels, respectively. For data display, the FRAP signal was normalized by dividing with V_1_+V_2_ obtained from the equations described below.

### Drugs

Glutamate, NMDA, ifenprodil and DHPG were dissolved in water; ionomycin in ethanol; KN-93, MK-801, thapsigargin and staurosporine in DMSO. All drugs were frozen in small aliquots at a concentration of at least 1000× and only thawed once. Glutamate was from Sigma, all others from Tocris Cookson Inc.

### Statistical analysis

All statistics and modeling was performed in R [27]. The model for the FRAP curves is described in the result section. The data was fitted to the model using least squares error minimization, i.e., the sum of the squares of the deviation of the data point and the predicted value was calculated and the parameters b, c, k1, V1, k2 and V2 were chosen to minimize this sum of squares. The t-tests, paired or unpaired, were performed with distinct variances in the two groups, i.e. without assuming equal variance in the two groups. The standard R function t-test was used. Data presented in the results section are given as average±standard deviation. Box plots show distribution and median.

### Electron microscopy analysis

Organotypic slices for transmission EM analysis were fixed in 1.5% paraformaldehyde and 1.5% glutaraldehyde in 0.1 M Sörensen's phosphate buffer (0.1 M NaH_2_PO_4_/Na_2_HPO_4_, pH 7.2) for 1 h followed by washing 3 times in Sörensen's phosphate buffer. Tissue was postfixed in 1% osmium tetroxid in Sörensen's phosphate buffer for 1 h, washed 3 times and dehydrated in ethanol with increasing concentration: 25, 50, 75 and 96% for 2×10 min respectively and 100% for 2×15 min. Prior to embedding, the slices were placed in 100% acetone for 2×20 min and then in a mixture of acetone and epon resin polybed 812 (Polysciences) 1∶1 over night. Next day the specimen was transferred to pure resin for at least 4 h before embedding in new pure resin and polymerization at 60°C for 48 h. The embedded specimen was sectioned in an ultratome (Super Nova, Reichert Jung) at 50 nm and mounted on slot copper grids previously covered with a thin film of pioloform. Grids were stained in 4% uranyl acetate for 30 min at 40°C and 0.5% lead citrate for 2 min at room temperature and observed with a Philips CM 10 electron microscope. In total, sections from 4 control and 4 glutamate treated slices were analyzed.

## Results

### ER fission occurs after stimulation of glutamate receptors

**Figure 1:**
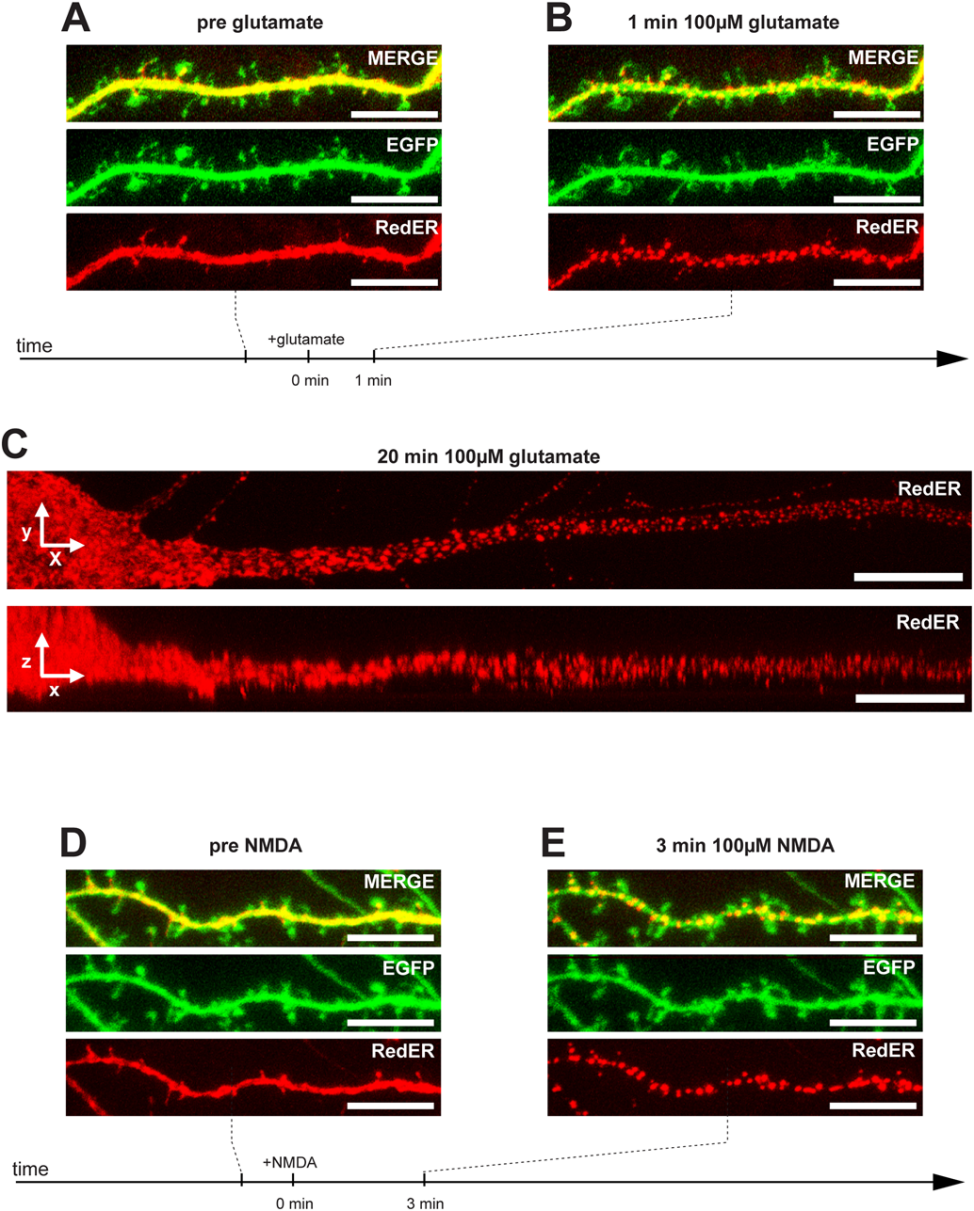
ER fission induced by glutamate or NMDA. (A) Representative image of a dendrite of a living hippocampal neuron transfected to express cytosolic EGFP and RedER showing normal ER morphology. (B) When exposed to 100 µM glutamate, rapid fission of the ER occurred after 1 min. Note the lack of change in dendritic and dendritic spine morphology in the green channel. (C) Image of proximal dendrite exposed to 100 µM glutamate for 20 min clearly showing the fragmented appearance of the ER in the xy (upper panel) and xz (lower panel) dimensions. (D) Representative image of a dendrite with normal morphology. (E) When exposed to 100 µM NMDA, rapid fission of the ER occurred within 3 min. Note the lack of change in dendritic and dendritic spine morphology in the green channel. Scale bar in all panels: 10 µm.

When examining primary hippocampal cultures transfected to express a fluorescent protein directed to the ER lumen (as described in e.g. [12]) we sometimes identified neurons with abnormal ER structure but with otherwise normal neuronal morphology. The ER in these cells was fragmented and existed as vesicles rather than the normal smooth and continuous appearance. To explore ER fragmentation further, we transfected hippocampal cultures to express a fluorescent protein targeted to the ER (EGFP-ER or DsRed2-ER (RedER) where the fluorescent protein is flanked by an ER-targeting sequence and a KDEL ER retention signal previously shown to label neuronal ER [28]–[30]) and a cytosolic fluorescent protein of another color (EGFP or DsRed2). When such cultures were treated with 100 µM glutamate at day in vitro 17 or later, the neuronal ER underwent rapid (within 1 to 10 min) fragmentation in 17 out of 19 neurons analyzed (Fig. 1A–C). The dramatic change in ER structure always preceded alterations in dendrite morphology. In most instances the ER of the whole neuron fragmented more or less simultaneously but in some cases a gradual distal to proximal wave of ER fission was noted (Fig. S1). Even concentrations of glutamate as low as 10 µM caused rapid fission of the ER in some neurons (data not shown). Selective stimulation of NMDA receptors with 100 µM NMDA had the same effect on ER structure as glutamate (Fig. 1D–E). After 10 min all 17 neurons had undergone ER fission. The ER vesicles found after glutamate as well as NMDA-induced fission were largely stationary and no evidence of trafficking of ER fragments within dendrites was obtained (not shown and Video S1 (between t = 6 and t = 11 min). Following 24 h of glutamate or NMDA all neurons were dead including those few where no ER fission was observed (data not shown).

### Modeling ER continuity

**Figure 2:**
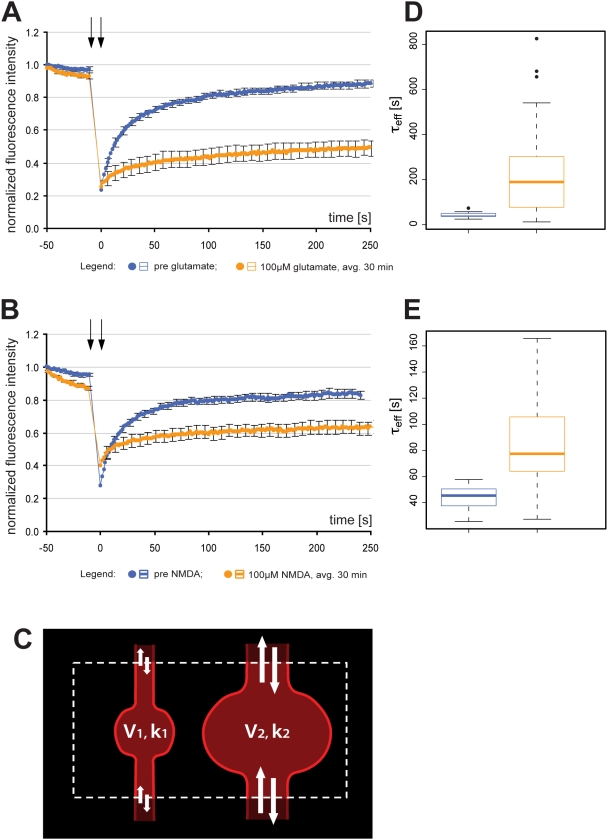
Analysis of ER fission by FRAP. (A and B) Normalized average FRAP signal over time in untreated neurons (blue) and the same neurons after (A) 100 µM glutamate, or (B) 100 µM NMDA (orange). Photobleaching was performed between the arrows. Time = 0 was set to when photobleaching ends and fluorescence starts to recover. Error bars are standard error of the mean (SEM). n = 19 for glutamate; n = 17 for NMDA. (C) The ER within the ROI (dashed line) was modeled as consisting of two volumes: V_1_ and V_2_. The RedER molecules move within these volumes with rate constants k_1_ and k_2_ respectively. (D and E) Box plot of τ_eff_ values of the same neurons shown in A and B. Untreated neurons are blue and the same neurons after (D) 100 µM glutamate or (E) NMDA are orange. Note the difference in scale between D and E. The line in the box is the median and the box represents the 25–75 percentiles. Whiskers extend to the extreme values as long as they are within a range of 1.5× box length. Values outside this range are plotted as outliers. avg.: average.

In order to determine whether the fragmented appearance of the ER really represented a break of ER continuity we performed FRAP experiments. For these experiments RedER was used rather than EGFP-ER as bleaching EGFP with the 488 argon laser bleaches DsRed2 and then the cytosolic signal cannot be used to control for focal drift (see methods). Multipolar and spiny neurons were selected and for each cell FRAP was recorded from a dendritic region of interest once prior to and once after the addition of glutamate or NMDA (Fig. S2). The time-point for the second FRAP recording varied for individual neurons between 1–55 minutes after the addition of drug. The reason for this variation in time was to permit collecting data from more than one neuron from each well. Our FRAP analysis clearly shows that ER with a fragmented appearance constitutes ER with broken or much reduced lumenal continuity after glutamate (Fig. 2A) or NMDA (Fig. 2B) treatment. To quantify FRAP results in a reliable way we decided to mathematically model ER continuity. The ER within the FRAP region of interest (ROI) was modeled as consisting of two compartments with volume V_1_ and V_2_, respectively (Fig. 2C). The compartments have rate constants, k_1_ and k_2_, respectively, defined as the rate at which a RedER molecule, bleached or unbleached, leaves the compartment and is replaced with a molecule from outside the ROI. Molecules within the ROI are bleached with the rate constant c during the photobleaching step. Our model also takes into account the lower level of photobleaching occurring during the recording scans by assigning the rate constant b to this bleaching. The equation governing the FRAP signal (S) during the recording scans in the compartment i = 1, 2 is:
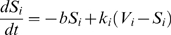
(1)


The equation during photobleaching is the same except for replacing b with c. In the time interval from t = 0 to t = t_1_, we record the signal, and hence the bleaching rate is b. The total signal is:
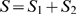



In the time interval t = t_1_ to t = t_2_, we perform the photobleaching with bleaching rate constant c. And finally, from time t = t_2_ and onwards, we record again with bleaching rate b. By solving these equations with the initial condition that all molecules are unbleached at time 0, i.e., S_i_ = V_i_, we get:
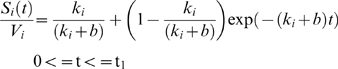
(2)

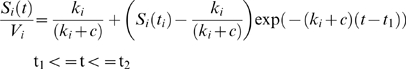
(3)

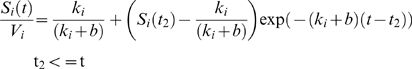
(4)


We fit this model to all the FRAP curves and it was able to fit the data very well (Fig. S3).

We wanted a single measure of ER continuity that could be used to compare the properties of the ER within neurons with different treatments and at different time-points. Such a measure can be made from the 6 parameters that describe the FRAP curve. The bleaching constants, b and c, however, are not intrinsic to the neurons, but depend on the laser power and other experimental settings. Hence, the continuity measure should be constructed from V_1_, V_2_, k_1_ and k_2_. With a single compartment, the rate constant, k, or the half-time of recovery τ = ln2/k, is a good measure. In this case we have two compartments, and hence a double exponential recovery, which does not have a simple half-time of recovery. In order to get a single recovery time, a time scale, T, needs to be defined. An effective rate constant, k_eff_, can then be defined by requiring that a single exponential recovery with rate k_eff_ at time T has the same recovery as the double exponential:
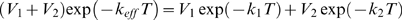



The effective half-time of recovery, which depends on the choice of time scale T, is given by:
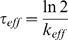



We fit the model to the experimental results from each individual neuron and τ_eff_ was calculated for T = 100 s. Glutamate treatment caused an increase in average τ_eff_ from 43.2±11.6 s to 256.2±242.7 s (n = 19, p<0.0001). For NMDA the increase was from 43.6±9.7 s to 87.4±38.4 s (n = 17, p<0.0001). The distribution of the data with median τ_eff_ for glutamate and NMDA is shown in Fig. 2D and Fig. 2E respectively. Average τ_eff_ after glutamate was significantly higher than after NMDA (p = 0.008).

### ER fission is reversible

**Figure 3:**
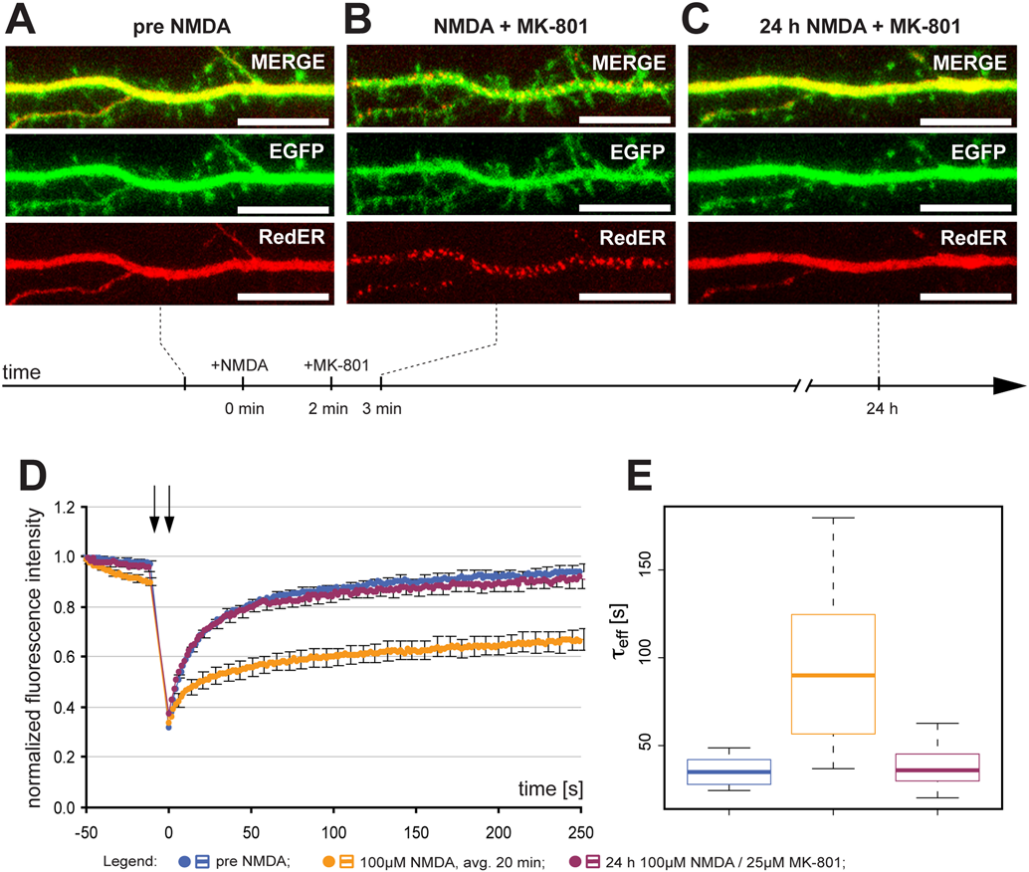
Reversibility of ER fission. (A) Representative image of a dendrite from a neuron with normal dendritic and ER structure. (B) 100 µM of NMDA caused rapid ER fission without any effect on gross dendritic structure. (C) Antagonizing NMDA receptor activation by 25 µM MK-801 allowed for ER fusion and recovery of ER structure by 24 h. (D) Normalized average FRAP signal over time in untreated neurons (blue), the same neurons after NMDA for an average of 20 min (orange) (note that MK-801 was added after 2–5 min) and after MK-801 for 24 h (purple). Photobleaching was performed between the arrows. Time = 0 was set to when photobleaching ends and fluorescence starts to recover. Error bars are SEM. n = 20. (E) Box plot of τ_eff_ values in untreated neurons (blue) and the same neurons after NMDA (orange) and MK-801 (purple). The line in the box is the median and the box represents the 25–75 percentiles. Whiskers extend to the extreme values as long as they are within a range of 1.5× box length. Scale bar in all panels: 10 µm. avg.: average.

We had previously observed that spontaneous fission could be followed by fusion (HT unpublished). The observed reversibility of fragmentation, along with the fact that neurons with fragmented ER often displayed normal neuronal morphology, makes it possible that such ER fission and fusion could have an important function in neurons. In order to further explore this aspect we established an experimental system where neuronal ER fission followed by fusion could be reliably induced without causing cell death. We found that inducing rapid ER fission with 100 µM NMDA followed by the addition of 25 µM of the NMDA receptor antagonist MK-801 2 to 5 minutes after NMDA, allowed ER fusion and cell survival after 24 h (Fig. 3A–C). In some instances ER fusion could be noted as early as 20 min after adding MK-801 (Video S1). After 24 h, ER and overall morphology of the neurons were as prior to NMDA exposure (Fig. 3A–C). Furthermore, we recorded FRAP at three time-points: prior to adding NMDA and MK-801, immediately after the drugs (5–60 min to allow collecting data from several cells in one well) and after 24 h (Fig. 3D). The observed fragmentation was found to correlate with an increase in average τ_eff_ from 35.1±8.0 s to 92.0±44.5 s (n = 20, p<0.0001). Furthermore, analysis of FRAP data revealed that at 24 h RedER fluorescence recovered from bleaching as prior to adding the drugs. The value for average τ_eff_ at 24 h was 37.6±11.3 s (p = 0.44 prior to drugs, p<0.0001 after fission). The distribution of the data with median τ_eff_ is shown in Fig. 3E.

### ER fission and fusion in hippocampal organotypic slices

**Figure 4:**
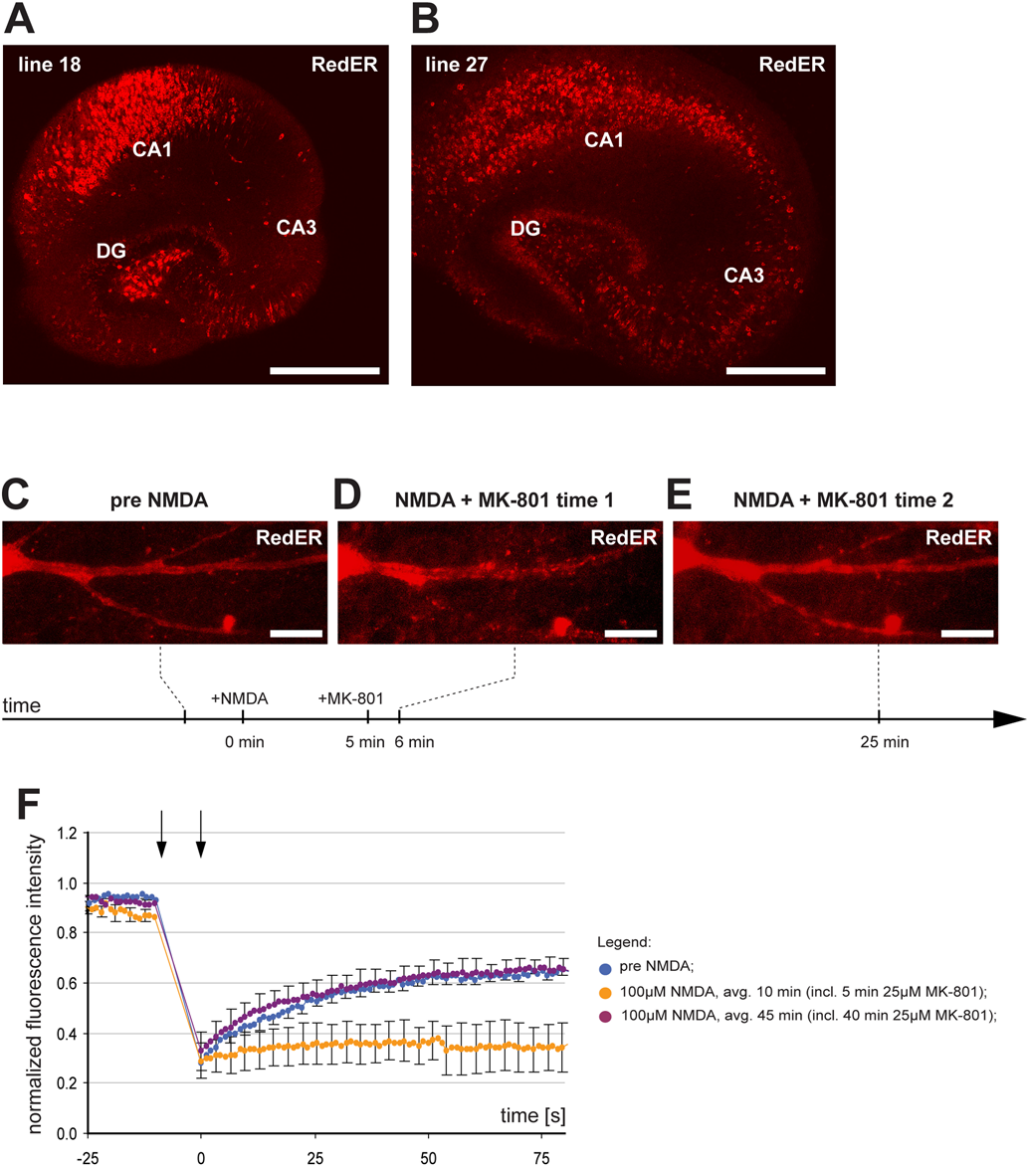
Reversible ER fission in organotypic slices. (A) Hippocampal expression of RedER in Thy1-RedER transgenic mouse line 18. (B) RedER expression in line 27. The expression pattern of the transgene differs slightly in that line 18 has no expression in pyramidal cells of CA3 but does express at high level in the hilus. (C) Representative images of dendritic ER structure in a CA3 pyramidal cell from line 27 with continuous ER prior to any treatment. (D) 100 µM NMDA triggered rapid ER fission. (E) 25 µM MK-801 led to fusion within 25 min. (F) Normalized average FRAP signal over time in untreated neurons (blue), the same neurons after NMDA for 10 min (orange) (note that MK-801 was added after 5 min) and after MK-801 (purple). Photobleaching was performed between the arrows. Time = 0 was set to when photobleaching ends and fluorescence starts to recover. Error bars are SEM. n = 4 neurons in 4 slices. Scale bars: 500 µm in A and B, 10 µm in C-E. avg.: average.

As the next step towards exploring the function of neuronal ER fission and fusion we generated transgenic mice expressing the RedER marker under the Thy1 promoter. Of the three founder lines tested, we chose to use lines 18 and 27 both of which showed high expression of the marker in the majority of pyramidal cells of the CA1. Line 27 showed additional RedER expression in a subset of CA3 pyramidal cells (Fig. 4A, B). We established hippocampal organotypic slices from these mice at postnatal day 7. In slices cultured for at least 10 days the ER of the soma and dendrites appeared smooth and continuous (Fig. 4C). Continuity of the dendritic ER lumen was confirmed by FRAP analysis (Fig. 4F and Video S2). Addition of glutamate or NMDA at 100 µM caused rapid ER fission that could be detected visually (Fig. 4D) as well as by FRAP (Fig. 4F and Video S3). Importantly, the same protocol that was developed to permit ER fusion in primary cultured neurons (i.e. 100 µM NMDA followed by 25 µM MK-801) had the same effect on neurons in slices (Fig. 4E, F) (4 out of 4 slices tested).

### Ultrastructure of ER fission

**Figure 5:**
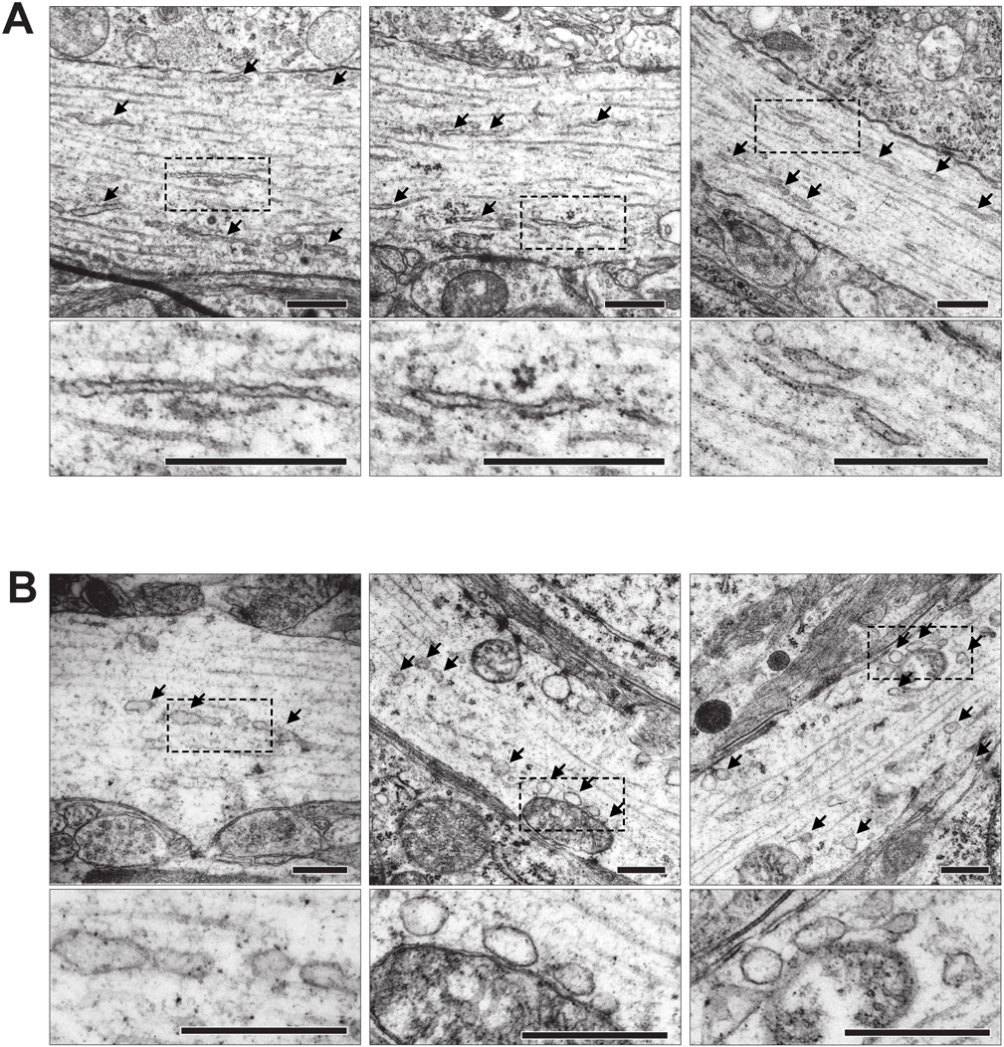
EM analysis of SER in dendrites. (A) Three representative images of dendrites from CA1 and CA3 in organotypic slices. ER profiles are indicated by arrows. (B) The ultrastructure after 100 µM glutamate for 5 min before fixing. No normal SER profiles were seen; instead the dendritic cytosol contains numerous dilated vesicles indicated by arrows. Boxed areas in the low magnification panel are enlarged below. Scale bar in all panels 500 nm.

**Figure 6:**
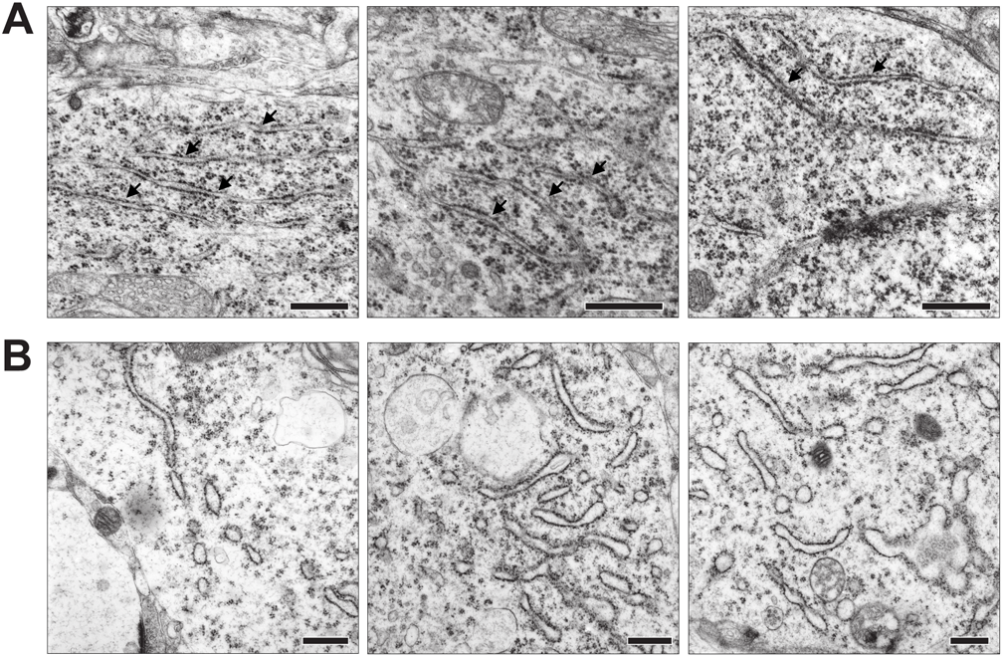
EM analysis of RER in neuronal somata. (A) Three representative images of CA1 and CA3 somata with normal ultrastructure. RER membranes studded with ribosomes are indicated with arrows. (B) The ultrastructure after 100 µM glutamate for 5 min. No normal RER profiles can be seen; instead the cytosol contains numerous dilated vesicles or tubules/cisterns that appear to have fewer ribosomes attached. Scale bar in all panels 500 nm.

While the optical methods used to assess ER structure demonstrate a clear reduction in ER lumenal continuity they cannot be used to determine the actual ultrastructural changes underlying the optical observations. We therefore examined ultrathin sections of organotypic slices with electron microscopy (EM). Untreated slices showed the expected ER structure with wavy membrane tubules and cisterns of smooth ER (SER) in the dendrites (Fig. 5A) and large cisterns of rough ER (RER) in the soma (Fig. 6A). In sections from slices treated with 100 µM glutamate none of the normal ER structures were observed. Within dendrites, normal SER morphology was absent. Instead, vesicles of varying sizes could be seen; often lined up giving the impression of being the result of a fragmentation event (Fig. 5B). In the soma the RER membranes were dilated, fewer ribosomes were attached and the membranes enclosed vesicles rather than the cisterns normally seen (Fig. 6B). EM analysis also revealed that ER in the CA3 region of line 18 (where the transgene is not expressed) was fragmented (not shown) demonstrating that ER fission is not caused by the presence of RedER in the ER.

### Mechanisms mediating glutamate induced ER fission

**Figure 7:**
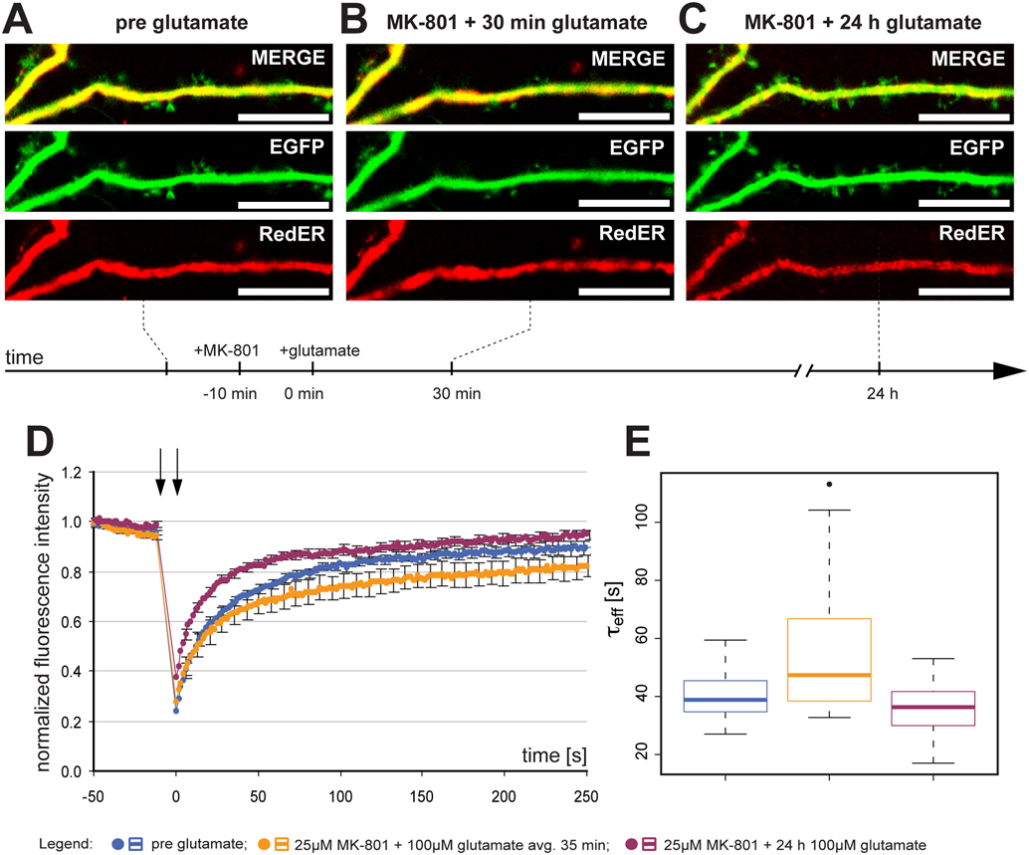
Inhibition of NMDA receptors is sufficient to block glutamate-induced ER fission. (A) Representative image of a dendrite from a neuron with normal dendritic and ER structure. (B) Treatment with 25 µM MK-801 for 10 min prior to 100 µM glutamate prevented ER fission although an effect on gross dendritic structure was seen (reduction in spine length). (C) After 24 h, 19 out of 20 cells had survived and resumed normal morphology. (D) Normalized average FRAP signal over time in untreated neurons (blue), the same neurons after MK-801 and glutamate for an average of 35 min (orange) and after 24 h (purple). Photobleaching was performed between the arrows. Time = 0 was set to when photobleaching ends and fluorescence starts to recover. Error bars are SEM. (E) Box plot of τ_eff_ values in untreated neurons (blue) and the same neurons after glutamate (orange). The line in the box is the median and the box represents the 25–75 percentiles. Whiskers extend to the extreme values as long as they are within a range of 1.5× box length. One neuron was outside this range and plotted as an outlier. Scale bars in all panels: 10 µm. avg.: average.

The fact that NMDA rapidly triggered ER fission led us to explore the role of the NMDA receptor. Primary cultures were treated with 25 µM MK-801 for 10 min followed by the addition of 100 µM glutamate. In this instance no fission was observed (Fig. 7A–C) although neuronal morphology was altered with blebbing of dendrites and reduced spine size. In spite of the lack of visible ER fragmentation, FRAP analysis (Fig. 7D) revealed a slight but significant increase in average τ_eff_ from 39.8±7.5 s before adding MK-801 and glutamate to 55.1±21.8 s (n = 20, p = 0.001) after 10–60 min of drug treatment. After 24 h 19 out of 20 cells had survived and average τ_eff_ was reduced to 36.3±9.5 s, that was not different from time-point one (p = 0.24) but significantly lower than after glutamate exposure (p = 0.008). The distribution of the data with median τ_eff_ is shown in Fig. 7E.

**Figure 8:**
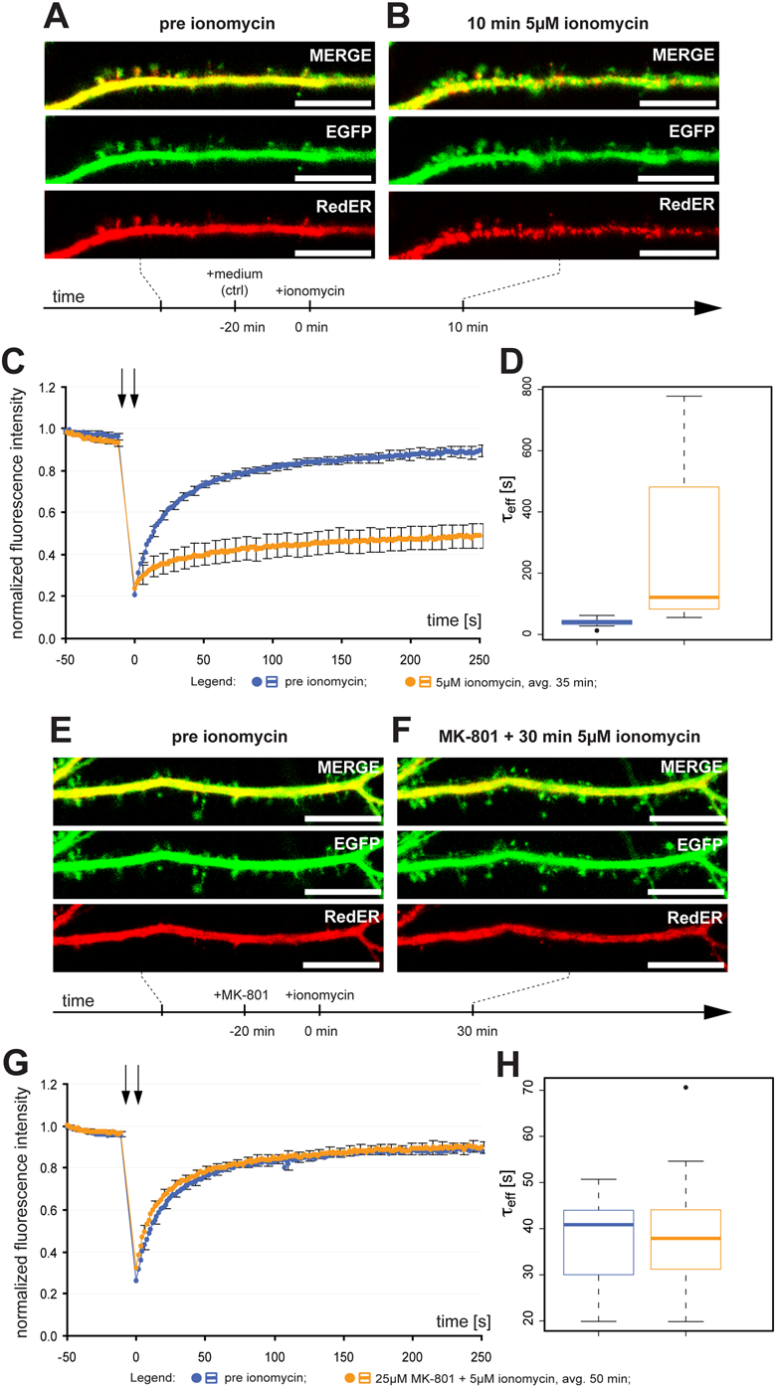
Ionomycin triggers NMDA receptor-mediated ER fission. (A) Representative image of a dendrite from a neuron with normal dendritic and ER structure. (B) Treatment with 5 µM ionomycin for 10 min caused ER fission along with a pronounced effect on gross dendritic structure (dendritic blebbing). (C) Normalized average FRAP signal over time in untreated neurons (blue) and the same neurons after ionomycin for an average of 35 min (orange). Photobleaching was performed between the arrows. Time = 0 was set to when photobleaching ends and fluorescence starts to recover. Error bars are SEM. n = 18. (D) Box plot of τ_eff_ values in untreated neurons (blue) and the same neurons after ionomycin (orange). The line in the box is the median and the box represents the 25–75 percentiles. Whiskers extend to the extreme values as long as they are within a range of 1.5× box length. (E) Representative image of a dendrite from a neuron with normal dendritic and ER structure. (F) Treatment with 25 µM MK-801 for 20 min prior to 5 µM ionomycin prevented ER fission and only caused minor effects on gross dendritic structure. (G) Normalized average FRAP signal over time in untreated neurons (blue) and the same neurons after MK-801 and ionomycin for an average of 50 min (orange). Photobleaching was performed between the arrows. Time = 0 was set to when photobleaching ends and fluorescence starts to recover. Error bars are SEM. n = 18. (H) Box plot of τ_eff_ values in untreated neurons (blue) and the same neurons after ionomycin (orange). The line in the box is the median and the box represents the 25–75 percentiles. Whiskers extend to the extreme values as long as they are within a range of 1.5× box length. One neuron was outside this range and plotted as an outlier. Scale bar in all panels: 10 µm. avg.: average.

The fact that no ER fission was seen when the NMDA receptor was blocked suggested to us that influx of calcium could be important for triggering ER fission. Indeed, the addition of 5 µM of the calcium ionophore ionomycin led to ER fission (Fig. 8A, B). FRAP analysis confirmed reduced protein mobility (Fig. 8C) and average τ_eff_ was increased from 39.5±11.1 s to 261.7±242.1 s (n = 18, p<0.0001). The distribution of the data with median τ_eff_ is shown in Fig. 8D. While such a result is typically interpreted as a general rise in cytosolic calcium is sufficient to trigger the phenomenon under study, secondary effects of ionomycin leading to NMDA receptor activation cannot be excluded. To test this, we incubated the cells with 25 µM MK-801 for 20 min prior to adding 5 µM ionomycin. Interestingly, when the NMDA receptor was blocked, ionomycin treatment did not trigger ER fission (Fig. 8E, F). FRAP experiments showed that MK-801 completely blocked the effect of ionomycin (Fig. 8G) with no change in average τ_eff_ before and after MK-801+ionomycin: 36.9±10.0 s and 38.3±12.1 s respectively (n = 18, p = 0.59). The distribution of the data with median τ_eff_ is shown in Fig. 8H.

An increase in cytosolic calcium can also be caused by release of ER calcium. However, neither type I metabotropic glutamate receptor activation with 100 µM 3,5-dihydrophenylglycine (DHPG) nor blockade of the sarco- and endoplasmic reticulum ATPase (SERCA) with 200 nM thapsigargin caused fission. NMDA-induced fission was independent of ER calcium levels as it occurred after thapsigargin (Table 1). Numerous downstream signaling events are triggered downstream of NMDA receptor activation. To learn more about the signaling pathways leading to activation of the hitherto unknown ER fission machinery we used pharmacological inhibition of the NMDA receptor as well as several enzymes known to be activated by the NMDA receptor to see if this affected the fission process. Ifenprodil is a non-competitive NMDA receptor antagonist but contrary to MK-801 it specifically inhibits activation of NR2B subunit-containing NMDA receptors. 10 µM ifenprodil added 10 min before NMDA did not inhibit ER fission (Table 1). 10 min of 20 µM staurosporine, a broad-spectrum kinase inhibitor was unable to block NMDA-induced ER fission (Table 1). As expected from the staurosporine result, specific inhibition of calcium and calmodulin regulated kinase II (CamKII) by 10 µM KN-93 had no effect on NMDA-induced ER fission (Table 1).

**Table 1:** Pharmacological manipulation of cytosolic calcium and calcium signaling.

n^a^	drug 1	effect 1 (n^b^)	drug 2	effect 2 (n^b^)
16	ifenprodil 10 µM/10 min	no ER fission (16)	NMDA 100 µM	ER fission (16)
20	thapsigargin 0.2 µM/60 min	no ER fission (20)	-	-
6	thapsigargin 0.2 µM/10 min	no ER fission (6)	NMDA 100 µM	ER fission (6)
10	KN-93 10 µM/10 min	no ER fission (10)	NMDA 100 µM	ER fission (10)
20	staurosporine 20 µM/20 min	no ER fission (20)	NMDA 100 µM	ER fission (20)
20	DHPG 100 µM/60 min	no ER fission (20)	-	-

Effect on ER structure.

a Total number of neurons tested.

b Number of neurons displaying the indicated response.

## Discussion

### Induction of neuronal ER fission

Elevated cytosolic calcium in non-neuronal cells was previously found to cause ER fission [21], [22]. Glutamate and NMDA trigger influx of extracellular calcium directly via the NMDA receptor and indirectly by activation of voltage-gated calcium channels. Release of ER calcium will occur by activation of metabotropic glutamate receptors and by calcium induced calcium release [1], [7]. Our experiments show that activation of the NMDA receptor is necessary and sufficient for inducing neuronal ER fission in dissociated and organotypic culture. First, glutamate-induced ER fission was inhibited by the NMDA receptor antagonist MK-801. Second, NMDA alone rapidly triggered ER fission. Third, we found that the calcium ionophore ionomycin caused ER fission but that, even in this case, ER fission required NMDA receptor activation. Finally, release of ER calcium triggered by activation of type I mGluRs or store depletion by thapsigargin did not lead to ER fission.

It should be noted that even if NMDA receptor activation was needed for obtaining optically defined ER fragments, glutamate treatment in the presence of the NMDA receptor antagonist MK-801 did cause a small but significant increase in τ_eff_. Taken together with the observation that glutamate caused a greater increase in τ_eff_ than NMDA; this may indicate that glutamate has some NMDA receptor-independent effect on ER structure. Nevertheless, we cannot exclude that the increase in τ_eff_ occurring after MK-801+glutamate treatment is a reflection of the rather dramatic but transient changes in neuronal morphology. Furthermore, the fact that τ_eff_ increases more after glutamate than after NMDA could still be an NMDA receptor-mediated effect as glutamate can be assumed to give stronger NMDA receptor activation than NMDA alone (glycine was not co-applied with NMDA).

The observed requirement for NMDA receptor activation to trigger ER fission raises the possibility that specific signaling pathways initiated at the NMDA receptor, rather than a general requirement for high cytosolic calcium, are important. Contrary to MK-801, ifenprodil (another non-competitive NMDA receptor antagonist) did not block NMDA induced ER fragmentation. Ifenprodil specifically inhibits NMDA receptors containing NR2B subunits [31]. Hence, activation of NR2A containing NMDA receptors is sufficient for induction of fission. Much of the signaling downstream of the NMDA receptor is mediated by kinases. However, staurosporine, a broad spectrum kinase inhibitor that blocks serine-threonine as well as tyrosine kinases [32] or the more specific CamKII inhibitor KN-93, did not affect NMDA-induced ER fission. Non-kinase calcium-induced signaling events downstream of the NMDA receptor are known. For instance, the small GTPases Ras and Rac can be activated by calcium-dependent guanine-nucleotide exchange factors that localize to spines [33], [34]. Alternatively, calcium sensitive parts of the hitherto unknown fission machinery may be localized close to NMDA receptors to induce fragmentation.

### Relevance of ER fission

Reorganization of the neuronal ER has been described previously but was not characterized in detail [9], [35]. Our results show distinct ER fission, that fragmented ER can fuse and we demonstrate that ER fission is not a point-of-no-return towards cell death. Hence, the poorly known mechanisms of ER fission and fusion are of direct relevance for understanding harmful processes and the development of neuroprotective therapeutic strategies. Indeed, the possibility of neuronal ER fission followed by fusion and cell survival has been suggested to occur after cerebral ischemia followed by reperfusion [36], [37]. Interestingly, the same conditions that cause ER fragmentation have been reported to induce stalling and fission of neuronal mitochondria. This process is known in greater detail than ER fission and mutations in several genes of the mitochondrial fission and fusion machinery are known to cause disease [38], [39].

While we can safely assume that regaining continuity of the ER is an absolute requirement for cellular survival, an important question remains: Is ER fission a harmful consequence of a harmful stimulus and hence the cell pays a price for ER fusion or is ER fission a protective response that helps the cell overcome a potentially harmful stimulus? The latter is supported by the fact that ER fission has been described in other cell types as a physiological phenomenon [19], [20] and we now report that neuronal ER fission occurs in the absence of morphological indicators of cell damage such as dendritic blebbing or loss of spines. What could then be the advantage for a cell to transiently fragment its ER? We hypothesize that the loss of continuity can be seen as creating bulkheads within the ER. Given that neuronal ER calcium can flow freely [40], [41], the partitioning caused by fission may serve to limit the release of calcium from the ER lumen under conditions when e.g. part of a neuron's dendritic arbor lies within a region of the brain parenchyma with excess levels of extracellular glutamate. It makes sense to limit release of ER calcium as either its contribution to elevated cytosolic calcium or the depletion of the ER store can contribute to neuronal damage [42]–[44]. ER fission may also serve to maintain damaged proteins within an area exposed to protein unfolding conditions. Potentially this sequestration of damaged proteins serves to prevent unnecessary induction of the unfolded protein response, which will lead to protein synthesis inhibition [2], [6].

The observation in non-neuronal cells that reversible ER fission is a structural correlate of physiological events on the cellular level [19], [20] suggests that a similar function in neurons cannot be excluded. Based on current knowledge, transient ER fission can be expected to alter the functional properties of the ER primarily by limiting the movement of ER restricted molecules and ions. Assuming that ER calcium channels function in the fragmented state, the pool of releasable calcium is dramatically altered when ER calcium tunneling [13] is aborted. Moreover, ER structural dynamics may have implications on local protein synthesis in dendrites, a process that has been implicated in synaptic plasticity [45]. In case ER vesicles are capable of supporting protein processing, the fragmented state will support highly localized and stationary protein synthesis, processing and export from the ER.

### The ultrastructure of ER fission

By light microscopic visual examination it appears that NMDA receptor stimulation causes the ER to fragment into isolated vesicles. FRAP analyses confirmed a reduction in ER protein mobility. Reduced mobility most likely also applies to calcium ions although presumably on a shorter time scale [40], [46]. Nevertheless, in only a few cases could we observe a near complete loss of FRAP (i.e. k_eff_ close to zero) when we bleached a dendrite with fragmented ER. Hence, from this data it cannot be excluded that rather than exclusively forming isolated fragments the ER may additionally form vesicles connected by thin tubules to resemble beads on a string. Data from EM studies of ER fission in other cell types have not provided conclusive evidence to rule out either complete vesiculation or beading of the ER [21], [47].

**Figure 9:**
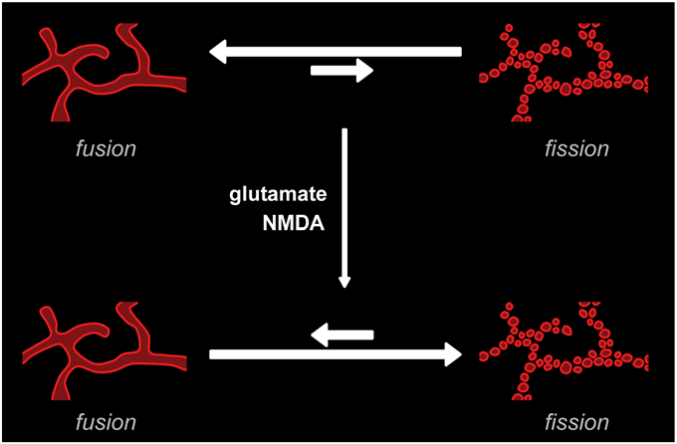
NMDA receptor activation may cause a shift in the balance between fission and fusion events. Rather than viewing the ER as having two static structural states (continuous and fragmented) the ER membranes may be undergoing constant fission and fusion events. In the resting state fusion events balance fission events. After NMDA receptor stimulation this balance may be shifted so that fusion events are more rare than fission events.

From our EM analysis of ultrastructural changes caused by short-term NMDA and glutamate treatment it can be concluded that somatic RER may indeed maintain connections between ER vesicles. In dendrites we found no evidence for connected ER fragments. However, one must bear in mind that on single sections thin connecting tubules may be missed and hence their existence cannot be ruled out. Thus, the physical basis for the low but existing FRAP after NMDA receptor activation still needs to be elucidated. We do not believe that it was caused by movement of large vesicles into the ROI; as such an event should easily be detected in our time-lapse images. Alternatively, some of the low level FRAP that we observed after fission could be caused by RedER molecules outside the ER proper, as the KDEL retention sequence allows trafficking out of the ER followed by cis-Golgi sorting back to the ER [48]. Additionally, ER vesicles may undergo cycles of fusion-fission and thus permit slow exchange of bleached molecules. Indeed, it can be hypothesized that, what is observed as fragmentation, is the outcome of a shift in the balance between constantly on-going fission and fusion events (Fig. 9).

## Supporting Information

Figure S1 Gradual distal to proximal ER fission. In neurons where ER fission did not occur instantaneously fragmentation always occurred gradually from the most distal parts of dendrites towards the soma. The image shows a neuron that was treated with 20 µM glutamate (5 min) and subsequently 25 µM MK801 to attenuate the stimulus. Scale bar: 10 µm.
(1.91 MB TIF)

Figure S2 Example of FRAP ROI. For all FRAP recordings a rectangular region of interest (ROI) was placed over a dendrite and a square ROI was placed immediately outside the dendrite to collect the background signal. The small boxes show the signal from the FRAP ROI. Scale bar: 10 µm (large image).
(2.20 MB TIF)

Figure S3 Curve fitting to data points. The FRAP recordings from the 19 neurons in the 100 µM glutamate experiment are shown. The upper panels are recordings prior to glutamate exposure and the lower panels are after 5–60 min of glutamate. The curve fit is in red.
(1.04 MB TIF)

Video S1 Reversibility of ER fission. Time-lapse recording of a neuron transfected to express RedER exposed to 20 µM glutamate at t = 5 min resulting in rapid ER fission. 25 µM MK-801 was added at t = 11 min and within 20 min the ER vesicles fused. Pixel dimensions: 151×303, frame rate: 25 fps, images collected at 1 Hz.
(8.31 MB MOV)

Video S2 FRAP experiment on untreated CA1 neuron. Time-lapse recording of a neuron in an organotypic slice expressing RedER under the Thy1 promoter (line 18). Bleaching was performed after the 5th frame (t = 24 s) in the ROI marked with a white rectangle. After bleaching fluorescence recovered rapidly indicating that the ER was continuous. This neuron was not included in the analysis. Pixel dimensions: 366×341, frame rate: 8 fps, images collected at 0.2 Hz.
(6.17 MB MOV)

Video S3 FRAP experiment on glutamate treated CA1 neuron. Time-lapse recording of a neuron in an organotypic slice expressing RedER under the Thy1 promoter (line 18). 100 µM glutamate was added 5 min prior to recording. Bleaching was performed after the 5th frame (t = 24 s) in the ROI marked with a white rectangle and a clear reduction in FRAP was noted. Pixel dimensions: 365×348, frame rate: 8 fps, images collected at 0.2 Hz.
(7.36 MB MOV)

## References

[1] Bardo S, Cavazzini MG, Emptage N (2006). The role of the endoplasmic reticulum Ca2+ store in the plasticity of central neurons.. Trends Pharmacol Sci.

[2] DeGracia DJ, Montie HL (2004). Cerebral ischemia and the unfolded protein response.. J Neurochem.

[3] Lindholm D, Wootz H, Korhonen L (2006). ER stress and neurodegenerative diseases.. Cell Death Differ.

[4] Mattson MP (2007). Calcium and neurodegeneration.. Aging Cell.

[5] Meldolesi J (2001). Rapidly exchanging Ca2+ stores in neurons: molecular, structural and functional properties.. Prog Neurobiol.

[6] Paschen W, Mengesdorf T (2005). Endoplasmic reticulum stress response and neurodegeneration.. Cell Calcium.

[7] Verkhratsky A (2005). Physiology and pathophysiology of the calcium store in the endoplasmic reticulum of neurons.. Physiol Rev.

[8] Zhang K, Kaufman RJ (2006). The unfolded protein response: a stress signaling pathway critical for health and disease.. Neurology.

[9] Terasaki M, Slater NT, Fein A, Schmidek A, Reese TS (1994). Continuous network of endoplasmic reticulum in cerebellar Purkinje neurons.. Proc Natl Acad Sci U S A.

[10] Martone ME, Zhang Y, Simpliciano VM, Carragher BO, Ellisman MH (1993). Three-dimensional visualization of the smooth endoplasmic reticulum in Purkinje cell dendrites.. J Neurosci.

[11] Spacek J, Harris KM (1997). Three-dimensional organization of smooth endoplasmic reticulum in hippocampal CA1 dendrites and dendritic spines of the immature and mature rat.. J Neurosci.

[12] Toresson H, Grant SG (2005). Dynamic distribution of endoplasmic reticulum in hippocampal neuron dendritic spines.. Eur J Neurosci.

[13] Petersen OH, Verkhratsky A (2007). Endoplasmic reticulum calcium tunnels integrate signalling in polarised cells.. Cell Calcium.

[14] Berridge MJ (1998). Neuronal calcium signaling.. Neuron.

[15] Pfeffer S (2003). Membrane domains in the secretory and endocytic pathways.. Cell.

[16] Shibata Y, Voeltz GK, Rapoport TA (2006). Rough sheets and smooth tubules.. Cell.

[17] Voeltz GK, Rolls MM, Rapoport TA (2002). Structural organization of the endoplasmic reticulum.. EMBO Rep.

[18] Antonny B, Schekman R (2001). ER export: public transportation by the COPII coach.. Curr Opin Cell Biol.

[19] Terasaki M, Jaffe LA (1991). Organization of the sea urchin egg endoplasmic reticulum and its reorganization at fertilization.. J Cell Biol.

[20] Terasaki M, Jaffe LA, Hunnicutt GR, Hammer JA (1996). Structural change of the endoplasmic reticulum during fertilization: evidence for loss of membrane continuity using the green fluorescent protein.. Dev Biol.

[21] Pedrosa Ribeiro CM, McKay RR, Hosoki E, Bird GS, Putney JW (2000). Effects of elevated cytoplasmic calcium and protein kinase C on endoplasmic reticulum structure and function in HEK293 cells.. Cell Calcium.

[22] Subramanian K, Meyer T (1997). Calcium-induced restructuring of nuclear envelope and endoplasmic reticulum calcium stores.. Cell.

[23] Dayel MJ, Hom EF, Verkman AS (1999). Diffusion of green fluorescent protein in the aqueous-phase lumen of endoplasmic reticulum.. Biophys J.

[24] Harmer AR, Gallacher DV, Smith PM (2002). Correlations between the functional integrity of the endoplasmic reticulum and polarized Ca2+ signalling in mouse lacrimal acinar cells: a role for inositol 1,3,4,5-tetrakisphosphate.. Biochem J.

[25] Caroni P (1997). Overexpression of growth-associated proteins in the neurons of adult transgenic mice.. J Neurosci Methods.

[26] Rytter A, Cronberg T, Asztely F, Nemali S, Wieloch T (2003). Mouse hippocampal organotypic tissue cultures exposed to in vitro “ischemia” show selective and delayed CA1 damage that is aggravated by glucose.. J Cereb Blood Flow Metab.

[27] Ihaka R, Gentleman R (1996). R: A language for data analysis and graphics.. Journal of Computational and Graphical Statistics.

[28] Jones VC, McKeown L, Verkhratsky A, Jones OT (2008). LV-pIN-KDEL: a novel lentiviral vector demonstrates the morphology, dynamics and continuity of the endoplasmic reticulum in live neurones.. BMC Neurosci.

[29] Ng AN, Toresson H (2008). Gamma-secretase and metalloproteinase activity regulate the distribution of endoplasmic reticulum to hippocampal neuron dendritic spines.. Faseb J.

[30] Horton AC, Ehlers MD (2003). Dual modes of endoplasmic reticulum-to-Golgi transport in dendrites revealed by live-cell imaging.. J Neurosci.

[31] Williams K, Russell SL, Shen YM, Molinoff PB (1993). Developmental switch in the expression of NMDA receptors occurs in vivo and in vitro.. Neuron.

[32] Ruegg UT, Burgess GM (1989). Staurosporine, K-252 and UCN-01: potent but nonspecific inhibitors of protein kinases.. Trends Pharmacol Sci.

[33] Farnsworth CL, Freshney NW, Rosen LB, Ghosh A, Greenberg ME (1995). Calcium activation of Ras mediated by neuronal exchange factor Ras-GRF.. Nature.

[34] Fam NP, Fan WT, Wang Z, Zhang LJ, Chen H (1997). Cloning and characterization of Ras-GRF2, a novel guanine nucleotide exchange factor for Ras.. Mol Cell Biol.

[35] Sokka AL, Putkonen N, Mudo G, Pryazhnikov E, Reijonen S (2007). Endoplasmic reticulum stress inhibition protects against excitotoxic neuronal injury in the rat brain.. J Neurosci.

[36] Petito CK, Pulsinelli WA (1984). Delayed neuronal recovery and neuronal death in rat hippocampus following severe cerebral ischemia: possible relationship to abnormalities in neuronal processes.. J Cereb Blood Flow Metab.

[37] Petito CK, Pulsinelli WA (1984). Sequential development of reversible and irreversible neuronal damage following cerebral ischemia.. J Neuropathol Exp Neurol.

[38] Tatsuta T, Langer T (2008). Quality control of mitochondria: protection against neurodegeneration and ageing.. Embo J.

[39] Cheung EC, McBride HM, Slack RS (2007). Mitochondrial dynamics in the regulation of neuronal cell death.. Apoptosis.

[40] Choi YM, Kim SH, Chung S, Uhm DY, Park MK (2006). Regional interaction of endoplasmic reticulum Ca2+ signals between soma and dendrites through rapid luminal Ca2+ diffusion.. J Neurosci.

[41] Solovyova N, Verkhratsky A (2003). Neuronal endoplasmic reticulum acts as a single functional Ca2+ store shared by ryanodine and inositol-1,4,5-trisphosphate receptors as revealed by intra-ER [Ca2+] recordings in single rat sensory neurones.. Pflugers Arch.

[42] Mattson MP, Zhu H, Yu J, Kindy MS (2000). Presenilin-1 mutation increases neuronal vulnerability to focal ischemia in vivo and to hypoxia and glucose deprivation in cell culture: involvement of perturbed calcium homeostasis.. J Neurosci.

[43] Wang C, Nguyen HN, Maguire JL, Perry DC (2002). Role of intracellular calcium stores in cell death from oxygen-glucose deprivation in a neuronal cell line.. J Cereb Blood Flow Metab.

[44] Wei H, Perry DC (1996). Dantrolene is cytoprotective in two models of neuronal cell death.. J Neurochem.

[45] Bramham CR, Wells DG (2007). Dendritic mRNA: transport, translation and function.. Nat Rev Neurosci.

[46] Park MK, Petersen OH, Tepikin AV (2000). The endoplasmic reticulum as one continuous Ca(2+) pool: visualization of rapid Ca(2+) movements and equilibration.. Embo J.

[47] Koch GL, Booth C, Wooding FB (1988). Dissociation and re-assembly of the endoplasmic reticulum in live cells.. J Cell Sci.

[48] Pelham HR (1988). Evidence that luminal ER proteins are sorted from secreted proteins in a post-ER compartment.. Embo J.

