# Supplementary figures and images for: NMDA Receptor Stimulation Induces Reversible Fission of the Neuronal Endoplasmic Reticulum
Source: PLoS One. 2009 Apr 21;4(4):e5250. doi: 10.1371/journal.pone.0005250 (PMC2668765; doi:10.1371/journal.pone.0005250)

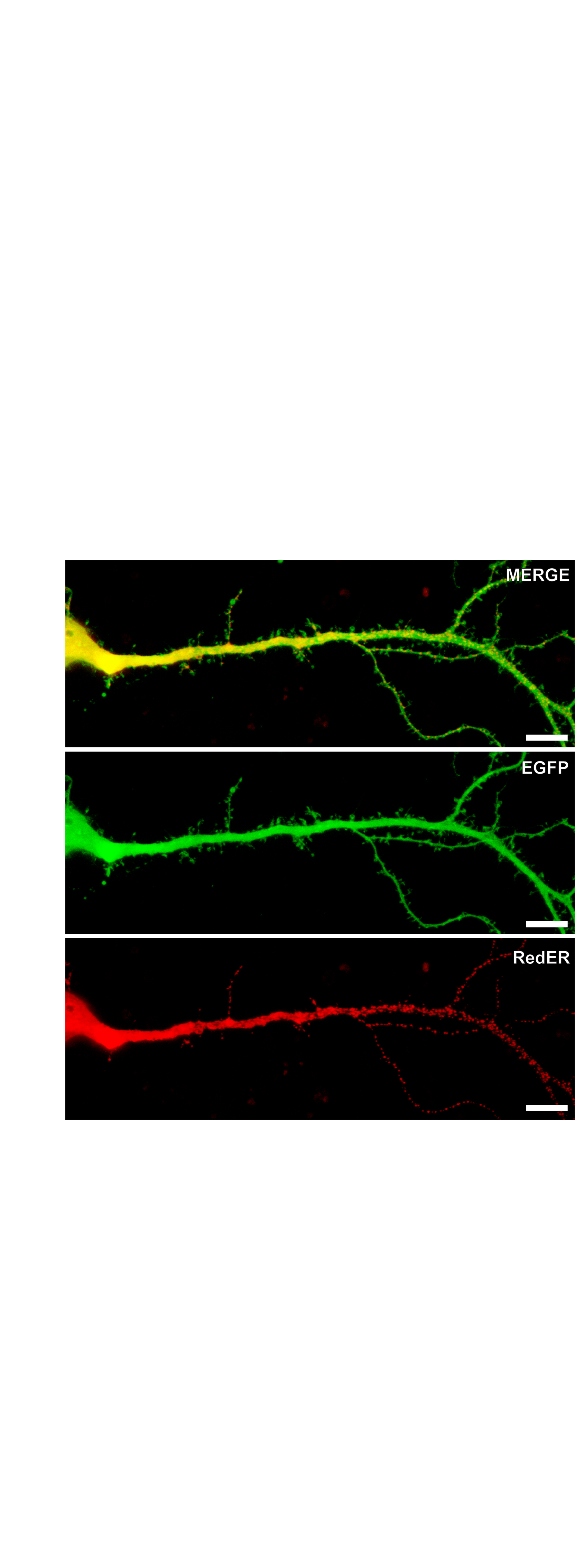

Supplement: Figure S1 — Gradual distal to proximal ER fission. In neurons where ER fission did not occur instantaneously fragmentation always occurred gradually from the most distal parts of dendrites towards the soma. The image shows a neuron that was treated with 20 µM glutamate (5 min) and subsequently 25 µM MK801 to attenuate the stimulus. Scale bar: 10 µm. (1.91 MB TIF) [file pone.0005250.s001.tif]

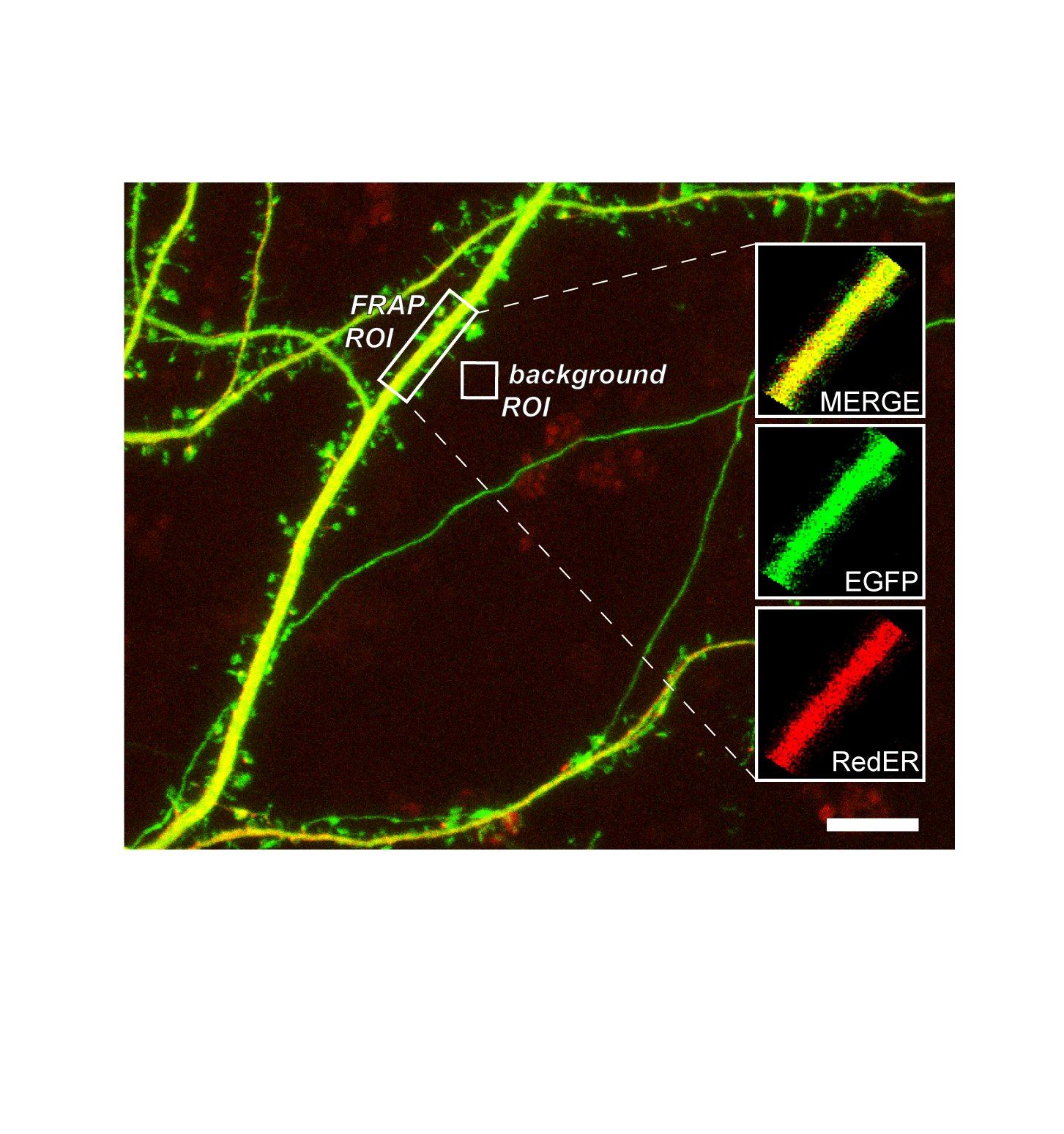

Supplement: Figure S2 — Example of FRAP ROI. For all FRAP recordings a rectangular region of interest (ROI) was placed over a dendrite and a square ROI was placed immediately outside the dendrite to collect the background signal. The small boxes show the signal from the FRAP ROI. Scale bar: 10 µm (large image). (2.20 MB TIF) [file pone.0005250.s002.tif]

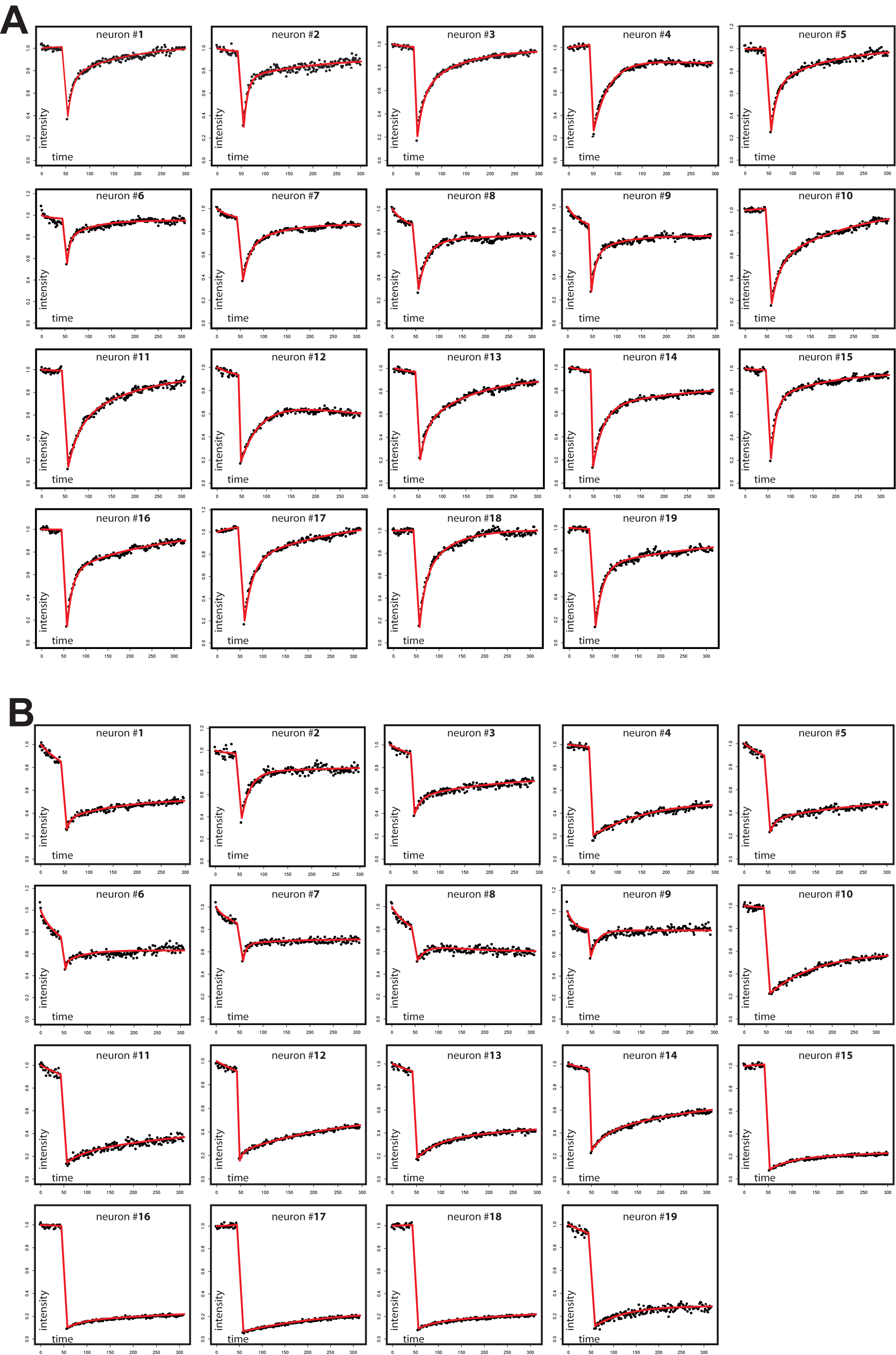

Supplement: Figure S3 — Curve fitting to data points. The FRAP recordings from the 19 neurons in the 100 µM glutamate experiment are shown. The upper panels are recordings prior to glutamate exposure and the lower panels are after 5–60 min of glutamate. The curve fit is in red. (1.04 MB TIF) [file pone.0005250.s003.tif]
